# Review of the Surgical Approaches for Peyronie's Disease: Corporeal Plication and Plaque Incision with Grafting

**DOI:** 10.1155/2008/263450

**Published:** 2008-11-04

**Authors:** Viet Q. Tran, Dennis H. Kim, Timothy F. Lesser, Sherif R. Aboseif

**Affiliations:** Section of NeuroUrology and Reconstructive Surgery, Department of Urology, Kaiser Permanente Medical Center, 4900 Sunset Boulevard, Los Angeles, CA 90027, USA

## Abstract

The understanding and management of Peyronie's disease (PD) has improved but elucidating the exact etiology of the disease has yet to be achieved. In this paper, we review the historical and clinical aspects of PD. We focus on the evolution of surgical management for PD and review recent published articles that compare popular surgical techniques such as plication and plaque incision with vein graft. These two techniques have been reported to be equivalent with respect to patient satisfaction; however, each technique has its own advantages and disadvantages.

## 1. INTRODUCTION

Peyronie's disease (PD) was named after the French physician Francois de la Peyronie in 1741. His original description was one of the fibrous cavernositis “preventing them from having normal ejaculation of semen” [1]. The disease is currently thought to affect between 3 and 9% of adult men, typically in the fifth to sixth decades of life. Overall, its prevalence appears to be rising, though this may be due to the fact that more men are seeking treatment for erectile dysfunction [2].

## 2. PATHOPHYSIOLOGY

Despite a volume of basic science and clinical research, much remains unknown about the etiology and ideal management of the disease. PD is a localized connective tissue disorder of the penis leading to fibrosis, scarring, and noncompliance of the tunica albuginea. One etiologic theory is that the root cause of the fibrosis is ischemia and inflammation from repeated penile trauma or microtrauma from activities such as sexual intercourse. The resulting microvascular tears in this region lead to collagen deposition in the form of plaques [3].

Some studies have suggested a link between PD and methotrexate, while others have shown a genetic predisposition to the disease due to an association with Dupuytren's contracture and with HLA-B7 and HLA-B27 antigens. Still other authors have identified circulating antibodies that may point to a possible immunologic cause of PD [4–7].

## 3. CLINICAL PRESENTATION

**Figure 1:**
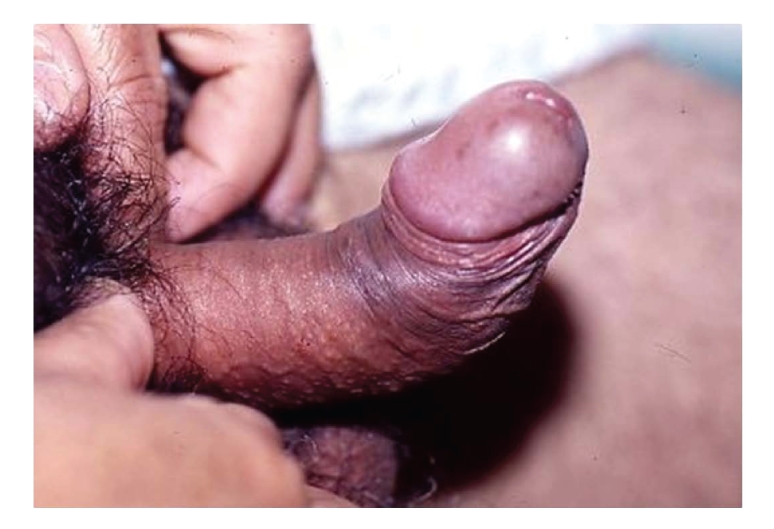
Penile deformity secondary to Peyronie's disease.

Patients typically present with three, occasionally simultaneous, chief complaints: a palpable plaque, a painful erection, and/or penile curvature. Penile curvature can in fact be so severe in that it interferes with the ability to engage in sexual intercourse (Figure 1). The disease undergoes a transition between two phases: an acute inflammatory phase and a chronic phase. Painful erections, developing penile curvature and nodule formation mark the acute inflammatory phase. This phase is self-limiting, typically lasting between six and eighteen months. Because the disease is evolving during this phase, the patient's pain, the degree of curvature, and the size of the plaque may also undergo change. The chronic phase is characterized by minimal or no pain with stable nodule size and degree of penile deformity [8].

## 4. TREATMENT

A variety of medical (i.e., nonsurgical) treatments with isolated reports of treatment “successes” have been published, but none have been substantiated in a randomized controlled fashion [9]. A thorough review of these therapies is beyond the scope of this article.

Surgical techniques for correcting the penile deformity from PD all share the same goals: correcting the curvature, preserving erectile function and penile length, and minimizing morbidity. For men with good erectile function, two main surgical concepts have been popularized: (1) lengthening the concave contracted side using a graft (with or without plaque incision/excision), and (2) shortening the convex, noncontracted side using tunical excision or plication. For men with poor erectile function and curvature, placement of a penile prosthesis to correct the erectile function can be sufficient if the degree of curvature is mild. If the curvature is more severe, a combination of one of the two approaches above with prosthesis placement is the preferred method of surgical repair.

### 4.1. The Nesbit procedure

The original tunical shortening procedure—the Nesbit procedure (named after the surgeon who first described it)—was initially applied to those with congenital penile curvature and later used for PD [10]. The method involved excising an ellipse of tunica on the side opposite the curvature, thereby straightening the penis. Though Nesbit's concepts have helped to guide the other surgical techniques, the Nesbit procedure itself is fraught with complications including penile shortening, recurrence of curvature, cavernous tissue herniation, and erectile dysfunction [11]. The Nesbit procedure is increasingly being replaced by one of the two surgical techniques described below.

### 4.2. Penile plication

**Figure 2:**
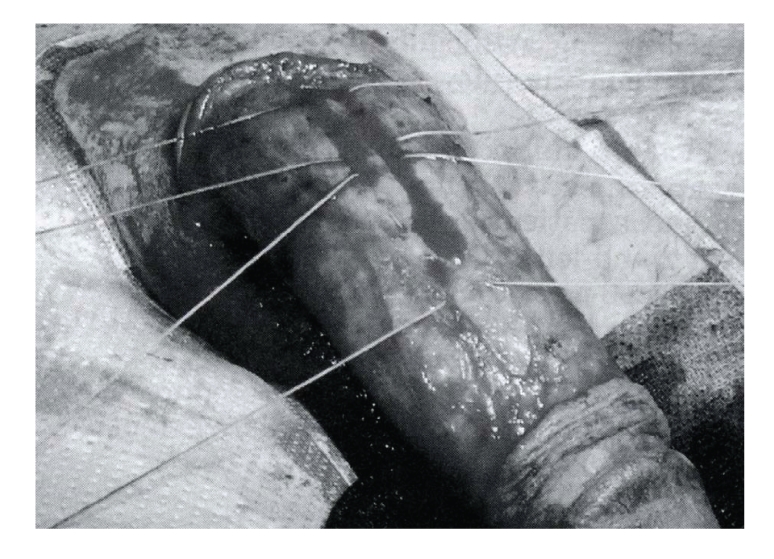
Correction of Peyronie's with corporeal plication.

Initially introduced by Essed and Schroeder as a less-invasive surgical option for PD, penile plication involves shortening the convex side of the curvature without excising the tunica (Figure 2) [12]. Lue expanded on this procedure by describing his “16 dot” technique that is rapid, involves no dissection of the neurovascular bundles or the urethra, spares the patient from tunical incision or excision, and reliably results in a straight penis in the appropriately selected patient. The procedure may be done with less potential morbidity to the patient under local anesthesia [13]. The relatively straightforward nature of the procedure, however, is balanced by its limited applicability to PD patients. Patients with bottleneck deformities, hourglass deformities, or lateral indentations are not appropriately treated with plication; in fact, the procedure is almost exclusively applicable to patients with simple curvature. It is the authors' experience, however, that the majority of patients with PD fit into this category.

Penile plication is associated with a number of well-described potential drawbacks that should be discussed in full detail with the patient. First, penile shortening has been reported from 41 to 90% of the time and is indeed the major drawback for most PD patients. PD patients with severe contraction may thus not be ideal candidates for plication; however, these patients often have an element of erectile dysfunction and may be better suited for receiving a penile implant. Second, sexual or erectile dysfunction associated with plication has been reported anywhere from 7 to 40% of the time in various studies. Other potential drawbacks described in previous studies include loss of penile sensation in 3–48% of patients and permanent palpable knots reported as “bothersome” in 12–18% of patients [13–16].

A study from 2007 reported patient-perceived outcomes from the plication procedure in 57 patients who had undergone the penile plication procedure for PD over a 10 year period [17]. With a median follow-up of 51 months, 90% reported a satisfactory cosmetic result, though only 71% reported a satisfactory functional result defined as a “straight or almost straight penis on erection with pain-free penetration and normal sexual intercourse.” Interestingly, the subset analysis of long-term patients pointed to excellent subjective durability of the plication procedure. 82% of these patients reported satisfactory cosmesis and 71% reported functional satisfaction. No objective data is presented, but, in our opinion, patient-perceived outcomes on the success, especially functionally, of the procedure should be of paramount importance when describing the outcomes of the procedure.

### 4.3. Plaque incision/excision with grafting

**Figure 3:**
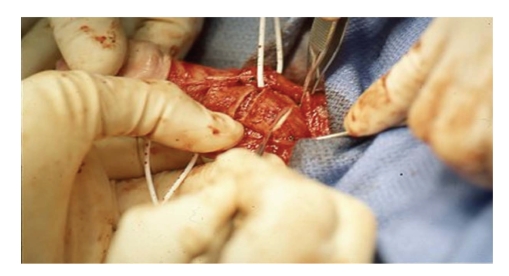
With the neurovascular bundles retracted laterally by the vessel loops, an H-incision is made at the point of maximal curvature.

Conceptually, plaque incision or excision with venous grafting approaches the contralateral aspect of the curvature—the concave side—with the aim to lengthen the curvature on that contracted side (Figure 3). Both incision and excision of the plaque with grafting have been described with similar results, but no studies have compared results from the two procedures. Additionally, the use of both autologous and synthetic grafts have been described, with the synthetic grafts reported as being less elastic with a potential predisposition toward wound infection [18]. Similar to the intracorporeal space, venous grafts are lined by vascular endothelium and are, therefore, theoretically more physiologic than other autologous tissues. Additionally, venous grafts provide excellent elasticity and durability. We prefer to use saphenous vein grafting because it is easy to harvest, reliably provides sufficient length and width, and is associated with little morbidity during harvest [19].

Side effects and complications of vein grafting procedures have also been well described. Initial results from the procedure have always been promising, with excellent patient satisfaction (86–92%) and high rates of penile straightening (59–96%) in the first 12 months. A recent study involving 70 patients treated with plaque incision with venous grafting with a mean follow-up of 41.7 months reported 53/70 patients (75.7%) with “completely straightened” penile curvature. Of the remaining patients, 12.8% had residual curvature less than 20 degrees and the remaining 11.4% had curvature more than 20 degrees [20]. Interestingly, the lack of durability of the results has led to a more cautious consideration of the applicability of tunical lengthening procedures in the urological community. Two studies presenting five-year follow-up data reported a lack of durability of the initial promising results with vein grafting. The first study identified a significant decrease in patient satisfaction due to either erectile dysfunction (22.5%) or penile shortening (35%) while the second study reported overall satisfaction falling from 86% initially to 60% at five years [21, 22]. One of the reasons cited in the latter study for a decrease in satisfaction was a subjective loss of penile length occurring in *all* of the patients at five years. Theoretically, tunical lengthening procedures should actually prevent or improve the penile shortening often associated with PD, but many men nonetheless complained of a reduction in penile length. Objective data from the same institution with 32 months of follow-up reported no change in mean pre-versus postoperative penile length, despite a patient-reported shortening in 40% of this group [23].

Clearly, the objective and patient reported assessment of penile length is incongruous, which should be considered when counseling patients on the “tunical lengthening” procedures. Additionally, the reported long-term results of the procedure have led to some skepticism in the urologic community about the durability of the procedure, which should be conveyed to the patient when describing the long-term outcomes.

### 4.4. Penile plication versus plaque incision with vein grafting

A recent study from our institution compared subjective patient reported experiences of tunical plication procedures (*n* = 35) with plaque incision and saphenous vein grafting procedures (*n* = 32) at one year of follow-up [24]. The short interval of follow-up is not ideal, especially with the questionable durability as described above, but nonetheless the results of our study are compelling. There were no statistically significant differences between the two groups with respect to straightening, overall patient satisfaction, erectile pain, and penile shortening. Patients who underwent plication were more likely to experience palpable sutures postoperatively but only 14% of patients reported this to be of a significant concern. Patients who underwent plaque incision with vein grafting were more likely to experience a loss in sensation as well as a loss in erectile rigidity. They were also more likely to be unable to have intercourse. Not surprisingly, the principle reason for the inability to have intercourse postoperatively was due to the loss in erectile rigidity. Loss in sensation was a significant patient concern in about one third of patients when it did occur. Length of operative times for the two groups varied drastically with an average time of 71 minutes for the plication group versus an average time of 234 minutes for the plaque incision and vein grafting group (*P* < .0001).

Based on the results of our study and the literature, at our institution we currently offer both procedures to patients with simple curvature secondary to PD. The literature has not clearly shown an advantage of one technique over the other in terms of long-term functional or cosmetic results. Indeed, recent literature has even pointed to a relative lack of functional durability of the vein grafting technique relative to plication, though this has never been demonstrated in a clinical trial or with objective data. All of this, as well as a description of the surgical technique, is described in detail when counseling patients pre-operatively.

In patients with more complex anatomic abnormalities due to PD (hourglass deformities, bottleneck deformities, or lateral indentations), we do not offer plication. These patients can clearly not be adequately treated with plication and require more extensive reconstructive and grafting technique.

## 5. CONCLUSION

The understanding and management of Peyronie's disease has been challenging but is improving. In reviewing the evolution of the surgical treatments, various refinements have evolved. This evolution has not led to one ideal surgical procedure which corrects all cases of PD, but rather there now exist a repertoire of surgical techniques that can be offered by urologists and selectively utilized for each individual's deformity. The surgical management of PD should always involve patient counseling of the different operative approaches and additionally should emphasize how these approaches will best meet patients' expectations. Being informed of the advantages and disadvantages of each surgical technique, patients are better able to make an informed decision.

## References

[1] de la Peyronie FG (1743). Sur quelques obstacles qui s'opposent à l'éjaculation naturelle de la semence. *Memoires de I'Academic Royale des Sciences Montpellier Chir*.

[2] Greenfield JM, Levine LA (2005). Peyronie's disease: etiology, epidemiology and medical treatment. *Urologic Clinics of North America*.

[3] Devine CJ, Somers KD, Jordan GH, Schlossberg SM (1997). Proposal: trauma as the cause of the Peyronie's lesion. *The Journal of Urology*.

[4] Phelan MJI, Riley PL, Lynch MP (1992). Methotrexate associated Peyronie's disease in the treatment of rheumatoid arthritis. *British Journal of Rheumatology*.

[5] Somers KD, Winters BA, Dawson DM (1987). Chromosome abnormalities in Peyronie's disease. *The Journal of Urology*.

[6] Nachtsheim DA, Rearden A (1996). Peyronie's disease is associated with an HLA class II antigen, HLA-DQ5, implying an autoimmune etiology. *The Journal of Urology*.

[7] Ralph DJ, Mirakian R, Pryor JP, Bottazzo GF (1996). The immunological features of Peyronie's disease. *The Journal of Urology*.

[8] Gelbard MK, Dorey F, James K (1990). The natural history of Peyronie's disease. *The Journal of Urology*.

[9] Taylor FL, Levine LA (2008). Non-surgical therapy of Peyronie's disease. *Asian Journal of Andrology*.

[10] Nesbit RM (1965). Congenital curvature of the phallus: report of three cases with description of corrective operation. *The Journal of Urology*.

[11] Andrews HO, Al-Akraa M, Pryor JP, Ralph DJ (2001). The Nesbit operation for Peyronie's disease: an analysis of the failures. *BJU International*.

[12] Essed E, Schroeder FH (1985). New surgical treatment for Peyronie's disease. *Urology*.

[13] Gholami SS, Lue TF (2002). Correction of penile curvature using the 16-dot plication technique: a review of 132 patients. *The Journal of Urology*.

[14] Chahal R, Gogoi NK, Sundaram SK, Weston PMT (2001). Corporal plication for penile curvature caused by Peyronie's disease: the patients' perspective. *BJU International*.

[15] Geertsen UA, Brok KE, Andersen B, Nielsen HV (1996). Peyronie curvature treated by plication of the penile fasciae. *British Journal of Urology*.

[16] Thiounn N, Missirliu A, Zerbib M (1998). Corporeal plication for surgical correction of penile curvature. Experience with 60 patients. *European Urology*.

[17] Fazili T, Kouriefs C, Anjum F, Masood S, Mufti GR (2007). Ten years outcome analysis of corporeal plication for Peyronie's disease. *International Urology and Nephrology*.

[18] Tornehl CK, Carson CC (2004). Surgical alternatives for treating Peyronie's disease. *BJU International*.

[19] Lue TF, El-Sakka AI (1998). Venous patch graft for Peyronie's disease—part I: technique. *The Journal of Urology*.

[20] Kadioglu A, Sanli O, Akman T, Cakan M, Erol B, Mamadov F (2008). Surgical treatment of Peyronie's disease: a single center experience with 145 patients. *European Urology*.

[21] Kalsi J, Minhas S, Christopher N, Ralph D (2005). The results of plaque incision and venous grafting (Lue procedure) to correct the penile deformity of Peyronie's disease. *BJU International*.

[22] Montorsi F, Salonia A, Briganti A Five year follow up of plaque incision and vein grafting for Peyronie's disease.

[23] Montorsi F, Salonia A, Maga T (2000). Evidence based assessment of long-term results of plaque incision and vein grafting for Peyronie's disease. *The Journal of Urology*.

[24] Kim DH, Lesser TF, Aboseif SR (2008). Subjective patient-reported experiences after surgery for Peyronie's disease: corporeal plication versus plaque incision with vein graft. *Urology*.

